# Supplementary material for: Phytochrome activates the plastid-encoded RNA polymerase for chloroplast biogenesis via nucleus-to-plastid signaling
Source: Nat Commun. 2019 Jun 14;10:2629. doi: 10.1038/s41467-019-10518-0 (PMC6570650; doi:10.1038/s41467-019-10518-0)
Supplement: Supplementary file 1 — Supplementary Information [file 41467_2019_10518_MOESM1_ESM.pdf]

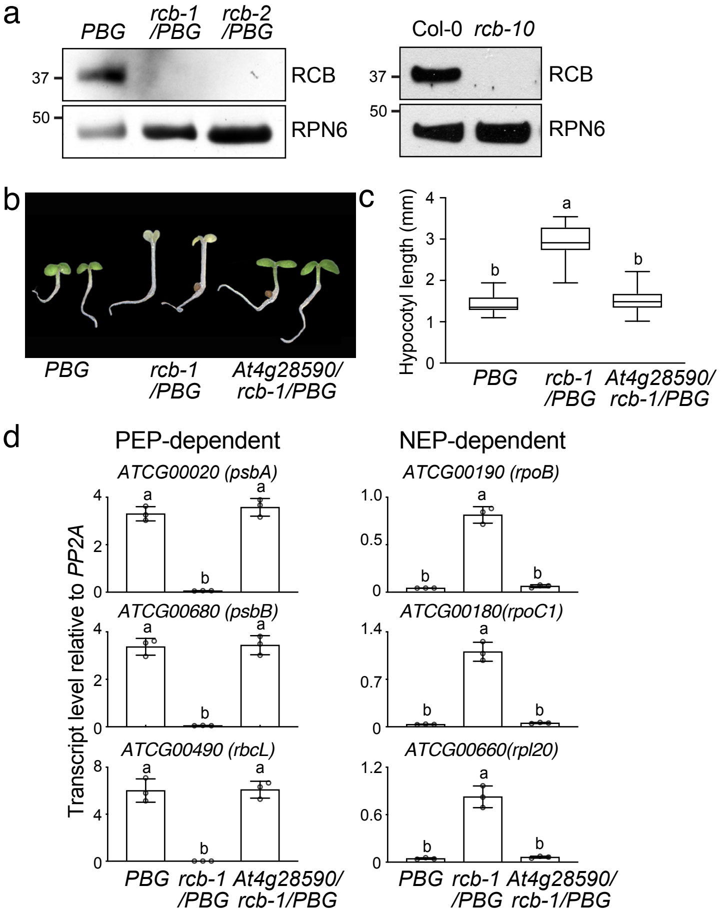

Supplementary Fig. 1. ***rcb-1/PBG*** **and** ***rcb-2/PBG*** **are null alleles and** ***rcb-1/PBG*** **can be rescued by expressing the cDNA of At4G28590. a**, *rcb-1/PBG*, *rcb-2/PBG*, and *rcb-10* are null alleles that fail to accumulate the gene product of At4G28590. Immunoblots showing the levels of the gene product of At4G28590 in 4-d-old *PBG*, *rcb-1/PBG*, *rcb-2/PBG*, Col-0, and *rcb-10* seedling grown in 10 μmol m^{-2} s^{-1} R light. The gene product of At4G28590 was detected by polyclonal antibodies raised against the gene product of At4G28590 (RCB antibodies), RPN6 was used as a loading control. **b**, Representative images of 4-d-old *PBG*, *rcb-1/PGB*, *At4G28590/rcb-1/PBG* seedlings grown in 10 μmol m^{-2} s^{-1} R light. *At4G28590/rcb-1/PBG* is a transgenic line expressing the cDNA of At4G28590 under the 35S promoter. **c**, The long hypocotyl phenotype of *rcb-1/PBG* was rescued in *At4G28590/rcb-1/PBG*. Box-and-whisker plots showing hypocotyl length measurements of seedlings shown in **b**. The boxes represent from the 25th to 75th percentiles, the bars equal the median values. Different letters denote statistically significant differences in hypocotyl length (ANOVA, Tukey's HSD, $p \leq 0.001$). **d**, The defects of *rcb-1/PBG* in plastidial gene expression were rescued in *At4G28590/rcb-1/PBG*. qRT-PCR results showing the transcript levels of representative PEP- and NEP-dependent genes in *PBG*, *rcb-1/PGB*, *At4G28590/rcb-1/PBG* seedlings grown in 10 μmol m^{-2} s^{-1} R light. Different letters denote statistically significant differences in transcript levels (ANOVA, Tukey's HSD, $p \leq 0.001$). The source data underlying the immunoblots in **a**, the hypocotyl measurements in **c**, and the qRT-PCR analysis in **d** are provided in the Source Data file.

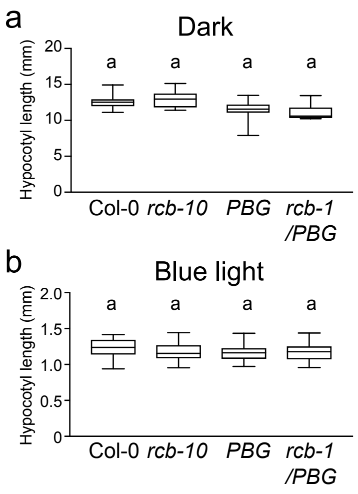

Supplementary Fig. 2. *rcb-10* and *rcb-1/PBG* exhibit normal hypocotyl responses in darkness and blue light.
a-b, Box-and-whisker plots showing hypocotyl length measurements of 4-d-old Col-0, *rcb-10*, *PBG*, and *rcb-1/PBG* seedlings grown in the dark (**a**) and in 10 μmol m^{-2} s^{-1} blue light (**b**). The boxes represent from the 25th to 75th percentiles, the bars represent the median values. Different letters denote statistically significant differences in hypocotyl length (ANOVA, Tukey's HSD, $p \leq 0.001$). The source data underlying the hypocotyl measurements in **a** and **b** are provided in the Source Data file.

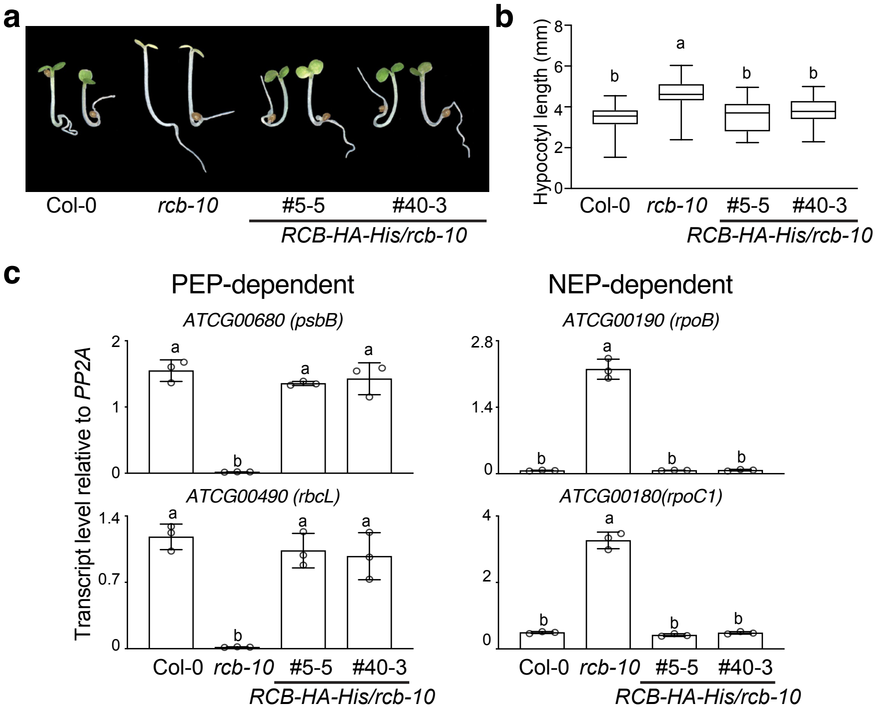

Supplementary Fig. 3. Expressing RCB-HA-His rescues the *rcb-10* mutant.
a, Representative images of 4-d-old Col-0, *rcb-10*, and *RCB-HA-His/rcb-10* (lines #5-5 and #40-3) seedlings grown in 10 μmol m^{-2} s^{-1} R light. **b**, *RCB-HA-His/rcb-10* lines rescued the long hypocotyl phenotype of the *rcb-10* mutant. Box-and-whisker plots showing hypocotyl length measurements of seedlings grown in **a**. The boxes represent from 25th to 75th percentiles, the bars equal the median values. Different letters denote statistically significant differences in hypocotyl length (ANOVA, Tukey's HSD, $p \leq 0.001$). **c**, The defects of *rcb-10* in plastidial gene expression were rescued in *RCB-HA-His/rcb-10*. qRT-PCR results showing the transcript levels of representative PEP- and NEP-dependent genes in 4-d-old Col-0, *rcb-10*, and *RCB-HA-His/rcb-10* (lines #5-5 and #40-3) seedlings grown in 10 μmol m^{-2} s^{-1} R light. Different letters denote statistically significant differences in mRNA levels (ANOVA, Tukey's HSD, $p \leq 0.001$). The source data underlying the hypocotyl measurements in **b** and the qRT-PCR analysis in **c** are provided in the Source Data file.

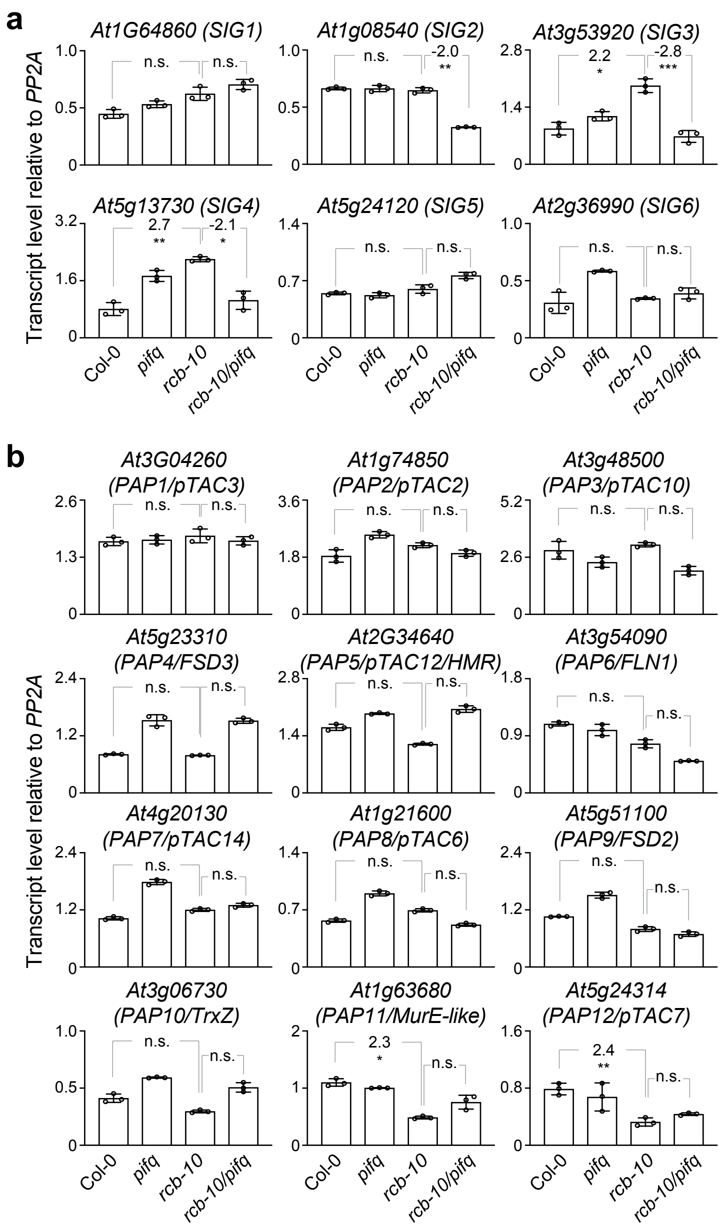

Supplementary Fig. 4. The transcript levels of nuclear-encoded PEP constituents are not significantly altered in *rcb-10* and *rcb-10/pifq* mutans.

a, qRT-PCR results showing the steady-state transcript levels of six sigma factors in 4-d-old Col-0, *pifq*, *rcb-10*, and *rcb-10/pifq* grown in 10 μmol m^{-2} s^{-1} R light. Fold changes are shown only in samples greater than two-fold changes between Col-0 and *rcb-10*, and *pifq* and *rcb-10/pifq* seedlings with statistical differences (*, p < 0.05; ** p < 0.01; *** p < 0.001; n.s., not statistically significant). Error bars represent SD of three biological replicates. **b**, qRT-PCR results showing the transcript levels of twelve PEP-associated proteins (PAPs) in 4-d-old Col-0, *pifq*, *rcb-10*, and *rcb-10/pifq* grown in 10 μmol m^{-2} s^{-1} R light. Fold changes are shown only in samples greater than two-fold changes between Col-0 and *rcb-10*, and *pifq* and *rcb-10/pifq* seedlings with statistical differences (*, p < 0.05; ** p < 0.01; *** p < 0.001; n.s., not statistically significant). Error bars represent SD of three biological replicates. The source data underlying the qRT-PCR analysis in **a** and **b** are provided in the Source Data file.

Supplementary Table 1. qRT-PCR primers for the nuclear genes examined in this study.

Accession	Gene name	Forward primer	Reverse primer
AT1G69960	*PP2A*	TATCGGATGACGATTCTTCGTGCAG	GCTTGGTCGACTATCGAATGAGAG
AT2G46970	*PIL1*	AAATTGCTCTCAGCCATTCGTGG	TTCTAAGTTTGAGGCGGACGCAG
AT4G16780	*ATHB2*	TCACAGTACTCTCAATCCGAAGC	CCGTAAGAACTCGCAGTCTAC
AT4G14130	*XTR7*	CACCGTCACTGCTTACTACTTG	CATTGGTGTGAAGAACATAAG
AT4G32280	*IAA29*	CACCATCATTGCCCGTATCA	CCACAGTAGCCGTTGTTGGA
AT1G64860	*SIG1*	AACTAAAACACGCAGCGAGGA	TCTTAAGGATCATTGCCTCCATTT
AT1G08540	*SIG2*	AGTCCAGAATGATAAGATTGCC	CTCTTTCACCCTATATGTTGCT
AT3G53920	*SIG3*	GTCTTTGTACATCCTCATCCT	TCTGCTTTCCTCTTTGACTG
AT5G13730	*SIG4*	CCATCTCCTTCTTTATCATCCC	CTATCAACCACTCTATCCACTG
AT5G24120	*SIG5*	GTGGTGAGGAGAAGAAAGTG	GCAATCCGTTTATCAAGACTC
AT2G36990	*SIG6*	CGGGCATTGTACAGGCTTAAG	AGCATGAAGTCCATGGCTGTTC
AT3G04260	*PAP1/pTAC3*	TCGCTGAGAAAGGAGCTAGG	GCGAGGATTTCTAGGCCTCT
AT1G74850	*PAP2/pTAC2*	TTGAACCGGACATGGAGACA	GGTGTTGAAAGCAACAAGCG
AT3G48500	*PAP3/pTAC10*	CATACCGGTTTCGGTTTCCC	CCTTCCTCCTCTGGCTTTGA
AT5G23310	*PAP4/FSD3*	CTGAACCACTTGGTGTCGTG	TTGGTTGGGACTTGGGACTT
AT2G34640	*PAP5/HMR*	CCAGTAATTGTATTGTGCAGAGAC	CACTTACATCACCATCTCCATC
AT3G54090	*PAP6/FLN1*	AGTCGCAAAGGGAGGAAGAA	TCGTCGTACGGAAACTCGAT
AT4G20130	*PAP7/pTAC14*	ATCGCTGCAGCAAGAACATT	TGCTCTGCTGTTGTGGGATA
AT1G21600	*PAP8/pTAC6*	AATGTCAAAGTCGCCGACAG	GTTCGGTGGTCACAGAATGG
AT5G51100	*PAP9/FSD2*	AGCCGGGAAACCTTGGATTA	CTCCCAGAAGAACTCGTGGT
AT3G06730	*PAP10/TrxZ*	GGAAGGTGCCGTTGATTGTT	GCGTGCAAACTCGTACTCAT
AT1G63680	*PAP11/MurE*	GCCGATGTTCACCCGTTAAA	AGAGGAGCTCCAACAGCAAT
AT5G24314	*PAP12/pTAC7*	TTGTGGTGAAAGGAGGTCGT	GGTCTCCAAGACCAATCCCA

Supplementary Table 2. Primers used for cDNA synthesis and qRT-PCR analysis of plastidial genes.

Accession	Gene name	Primer for cDNA synthesis	Primer pair for qRT-PCR
ATCG00020	*psbA*	TAGATGGAGCCTCAACAGCAGCTA	ACATTTCTTCTTAGCGGCTT<br>CGTCCTTGACTATCAACTACTGA
ATCG00680	*psbB*	CATCCAAATCTGGATCAATACCAG	ACATTTCTTCTTAGCGGCTT<br>CGTCCTTGACTATCAACTACTGA
ATCG00490	*rbcL*	CTTCACAAGCAGCAGCTAGTTCAGG	GGAGATGATTCTGTACTACAAT<br>GTCCCTCATTACGAGCTTGTAC
ATCG00190	*rpoB*	CAATGATAGTGGTACCAAGTACTTC	CTAGTGGACATTATGCACTTGT<br>CAGATTTATAAGTAAGCATCTCTTG
ATCG00180	*rpoC1*	GTATAGCTTCCTCGATTTCTCG	GATGCAATTGGAGCTTATCG<br>CGATAGGAACTTCTCTTGAAGC
ATCG00660	*rpl20*	TGTGCAAGTATTTTCCGATTAAG	GAGCTTTAGTTTCGGCTCATC<br>AATTACGGCATTTATTCGAGTG

Supplementary Table 3. Primers used for making constructs.

Accession	Gene name	Vector	Forward primer	Reverse primer
AT4G28590	*RCB*	pCHF1	CGGGAGCTCATGAGTTTCTTCGCTGTTGC	CGGGGTACCCTAACAGTACGGGGTTACATTAG
AT4G28590	*RCB-HA-His*	pCHF1-HA-His	CGGGAGCTCATGAGTTTCTTCGCTGTTGC	CGGGGTACCACAGTACGGGGTTACATTAGC
AT4G28590	*RCB*	pET42b	CGGGGATCCGATGAGTTTCTTCGCTGTTGC	CGGCTGCAGCTAACAGTACGGGGTTACATTAG
AT4G28590	*RCB-antigen*	pET42b	GGCGGATCCGAAATCGAAGCGTGGGCGGCGG	GGCCTGCAGTCATGGACTAGGATGAAAAGCAAAG
AT4G28590	*RCB-FL*	pCMX-PL2	CGGCTGCAGATGAGTTTCTTCGCTGTTGC	CGGGGATCCCTAACAGTACGGGGTTACATTAG
AT4G28590	*RCB-Δ10*	pCMX-PL2	GCTTGATATCGAATTCCTGCAATGCCAAGATCTTCTATGCTTC	ACTAGCTAGCTGGCCAGCTAACAGTACGGGGTTACAT
AT4G28590	*RCB-Δ20*	pCMX-PL2	GCTTGATATCGAATTCCTGCAATGAATTCGAGCTTCTCTGATATG	ACTAGCTAGCTGGCCAGCTAACAGTACGGGGTTACAT
AT4G28590	*RCB-Δ30*	pCMX-PL2	GCTTGATATCGAATTCCTGCAATGCCACTATTTGTTTTCCCGG	ACTAGCTAGCTGGCCAGCTAACAGTACGGGGTTACAT
AT4G28590	*RCB-Δ40*	pCMX-PL2	GCTTGATATCGAATTCCTGCAATGTCCCGGAGCGTGAAACG	ACTAGCTAGCTGGCCAGCTAACAGTACGGGGTTACAT
AT4G28590	*RCB-Δ51*	pCMX-PL2	CGGCTGCAGATGTCTGATTCCGTACTAGACC	ACTAGCTAGCTGGCCAGCTAACAGTACGGGGTTACAT
AT4G28590	*RCB-Δ92*	pCMX-PL2	GGCCTGCAGATGAAATCGAAGCGTGGGCGG	CGGGGATCCCTAACAGTACGGGGTTACATTAG
