# Supplementary material for: Phytochrome activates the plastid-encoded RNA polymerase for chloroplast biogenesis via nucleus-to-plastid signaling
Source: Nat Commun. 2019 Jun 14;10:2629. doi: 10.1038/s41467-019-10518-0 (PMC6570650; doi:10.1038/s41467-019-10518-0)
Supplement: Supplementary file 3 — Description of Additional Supplementary Files [file 41467_2019_10518_MOESM3_ESM.pdf]

Description of Additional Supplementary Files

File Name: Supplementary Data 1
Description: . SSTF (statistically significantly and by two-fold) genes between 4-d old rcb-10 and Col-0 (WT) seedlings grown under continuous 10 µmol m-2 s-1 R light. SSTF genes are defined as: (a) greater than two-fold difference in expression values between two genotypes and (b) the difference was statistically significant ($p \leq 0.05$) using a t-test adjusted for the false discovery rate (FDR).

File Name: Supplementary Data 2
Description: RCB-dependent genes are defined as the SSTF genes between rcb-10-R and WT-R (992 genes, Supplementary Data 1). HMR-dependent genes are the SSTF genes between hmr-5-R and WT-R (1348 genes) 57 .

File Name: Supplementary Data 3
Description: The PIF direct target genes have been defined previously58 . The raw expression data of the listed genes in Col-0 (WT) and rcb-10, as well as their fold changes in rcb-10, are shown. The columns “final target class” and “PIF regulation” are from Supplementary Table 4 of Pfeiffer et al. (2014) 58 .

File Name: Supplementary Data 4
Description: A list of PIF direct target genes (from Supplementary Data 3) that were changed statistically significantly ($p \leq 0.05$) and by 1.5-fold in 4-d old rcb-10 and hmr-5 compared with Col-0 (WT).
